# Ventricular Septal Rupture Complicating Silent Myocardial Infarction

**DOI:** 10.14797/mdcvj.1168

**Published:** 2022-12-06

**Authors:** Amr Telmesani, Qasim Al Abri, Mohammed Chamsi-Pasha

**Affiliations:** 1 Houston Methodist DeBakey Heart & Vascular Center, Houston Methodist Hospital, Houston, Texas, US; 2 Department of Medicine, Umm Al-Qura University, Makkah, Saudi Arabia

**Keywords:** ventricular septal rupture, myocardial infarction, intracardiac shunting

## Abstract

A 55-year-old gentleman presented to the emergency department with shortness of breath for the past 3 days. Cardiac magnetic resonance imaging assessed intracardiac shunting and a mechanism of ventricular septal rupture (VSR), showing significant left-to-right shunting and Qp:Qs of 4:1. There was transmural myocardial infarction as well as an aneurysm at the diaphragmatic inferior wall of the left ventricle.

A 55-year-old gentleman with no significant past medical history except cigarette smoking presented to the emergency department with shortness of breath for the past 3 days. No associated symptoms of chest pain were reported. Upon arrival, he was normotensive with normal oxygen saturation on room air. An electrocardiogram showed sinus rhythm with inferior Q waves. Blood work showed elevated troponin and brain natriuretic peptide (BNP). A chest X-ray showed pulmonary edema. A transthoracic echocardiogram demonstrated large inferoseptal ventricular septal rupture (VSR) with left-to-right shunt and hyperdynamic left ventricle (Figure 1 A, B; Videos 1, 2). Cardiac magnetic resonance imaging followed to assess intracardiac shunting and a mechanism of VSR, showing significant left-to-right shunting and Qp:Qs of 4:1. There was transmural myocardial infarction (Figure 1 C, D; Video 3) as well as an aneurysm at the diaphragmatic inferior wall of the left ventricle (LV) (Figure 1 E). Subsequent left heart catheterization showed complete occlusion of the right coronary artery and severe stenosis of obtuse marginal 1 (OM1). An intra-aortic balloon pump was inserted in anticipation of surgery. Intraoperatively, there was extensive necrosis of the entire inferior LV wall and inferior septum with a 2.5-cm septal defect near the base of the heart. The patient underwent exclusion patch repair with bovine pericardium of VSR and coronary artery bypass grafting to OM1 with extracorporeal membrane oxygenation support. He underwent cardiac computed tomography, which showed a small residual VSD (Figure 1 F, G; Video 4). He eventually recovered after a long hospital stay and was discharged home.

**Figure 1:**
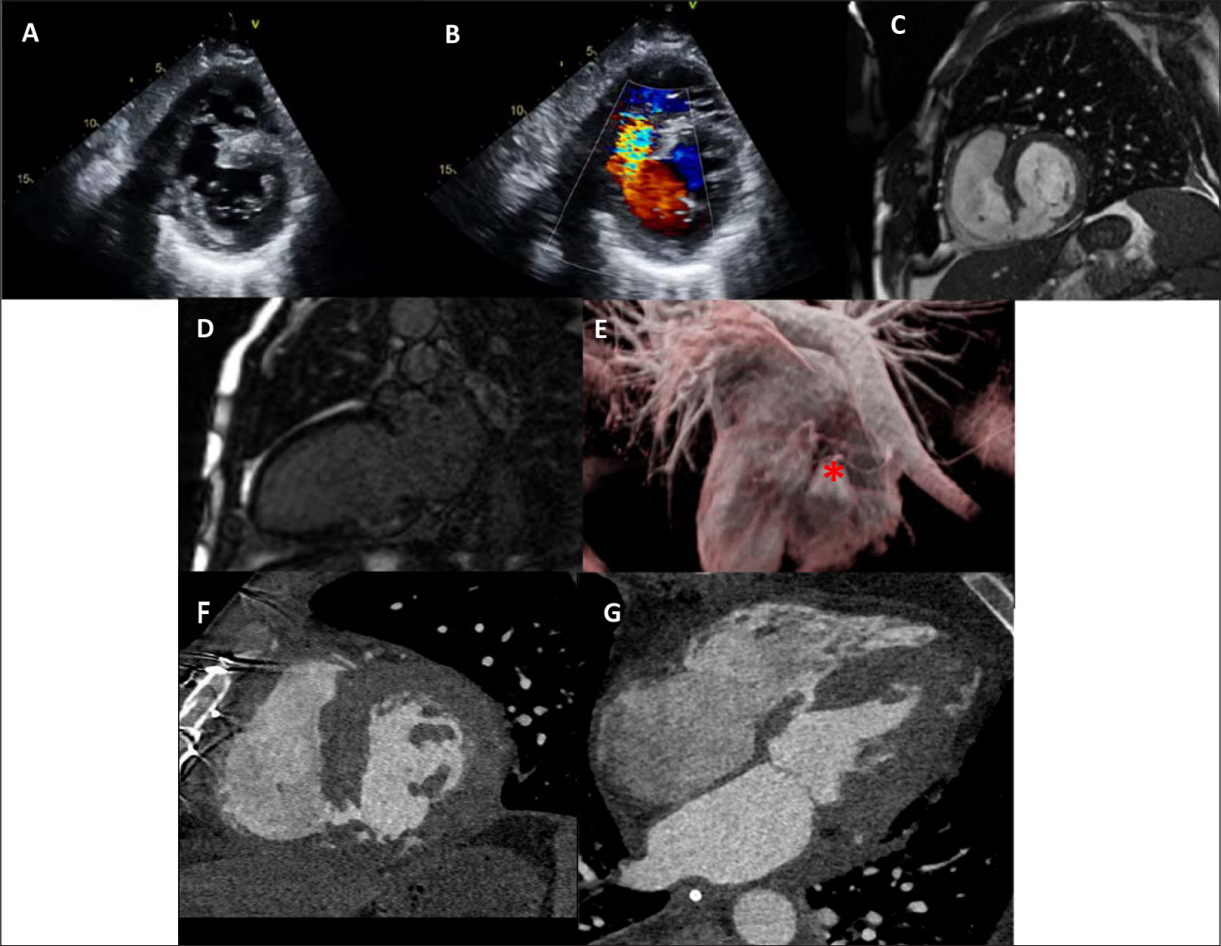
**(A)** Transthoracic echocardiogram of basal short-axis view showing inferoseptal ventricular septal rupture (VSR) and **(B)** color Doppler of inferoseptal VSR with left-to-right shunt. **(C)** Cardiac magnetic resonance imaging (CMR) of basal short-axis view showing inferoseptal VSR and **(D)** 2-chamber view of nonviable basal inferior wall. **(E)** Cardiac magnetic resonance of 3-dimensional reconstruction showing inferior aspect of the heart with inferior wall aneurysm (red asterisk). **(F)** Computed tomography of residual ventricular septal defect showing short-axis view and **(G)** 4-chamber view.

**Video 1:** Transthoracic echocardiogram, 2D apical four-chamber view, short-axis shows ventricular septal rupture, see also at https://youtu.be/Lqozswl-tF0.

**Video 2:** Transthoracic echocardiogram, color Doppler apical four-chamber view, shows ventricular septal rupture left-to-right shunt, see also at https://youtu.be/uMbyqaZt70I.

**Video 3:** Cardiac magnetic resonance imaging, cine short-axis view, shows ventricular septal rupture, see also at https://youtube.com/shorts/sCPeIJL-TCw.

**Video 4:** Cardiac magnetic resonance imaging, velocity encoding short-axis view, shows ventricular septal rupture defect, see also at https://youtube.com/shorts/iD4u8PXWMyk.

